# Molecular Fingerprints of Hemoglobin on a Nanofilm Chip

**DOI:** 10.3390/s18093016

**Published:** 2018-09-09

**Authors:** Yeşeren Saylan, Adil Denizli

**Affiliations:** Department of Chemistry, Hacettepe University, 06800 Ankara, Turkey; yeseren@hacettepe.edu.tr

**Keywords:** chip, hemoglobin, molecular imprinting, nanofilm, surface plasmon resonance

## Abstract

Hemoglobin is an iron carrying protein in erythrocytes and also an essential element to transfer oxygen from the lungs to the tissues. Abnormalities in hemoglobin concentration are closely correlated with health status and many diseases, including thalassemia, anemia, leukemia, heart disease, and excessive loss of blood. Particularly in resource-constrained settings existing blood analyzers are not readily applicable due to the need for high-level instrumentation and skilled personnel, thereby inexpensive, easy-to-use, and reliable detection methods are needed. Herein, a molecular fingerprints of hemoglobin on a nanofilm chip was obtained for real-time, sensitive, and selective hemoglobin detection using a surface plasmon resonance system. Briefly, through the photopolymerization technique, a template (hemoglobin) was imprinted on a monomeric (acrylamide) nanofilm on-chip using a cross-linker (methylenebisacrylamide) and an initiator-activator pair (ammonium persulfate-tetramethylethylenediamine). The molecularly imprinted nanofilm on-chip was characterized by atomic force microscopy and ellipsometry, followed by benchmarking detection performance of hemoglobin concentrations from 0.0005 mg mL^−1^ to 1.0 mg mL^−1^. Theoretical calculations and real-time detection implied that the molecularly imprinted nanofilm on-chip was able to detect as little as 0.00035 mg mL^−1^ of hemoglobin. In addition, the experimental results of hemoglobin detection on the chip well-fitted with the Langmuir adsorption isotherm model with high correlation coefficient (0.99) and association and dissociation coefficients (39.1 mL mg^−1^ and 0.03 mg mL^−1^) suggesting a monolayer binding characteristic. Assessments on selectivity, reusability and storage stability indicated that the presented chip is an alternative approach to current hemoglobin-targeted assays in low-resource regions, as well as antibody-based detection procedures in the field. In the future, this molecularly imprinted nanofilm on-chip can easily be integrated with portable plasmonic detectors, improving its access to these regions, as well as it can be tailored to detect other proteins and biomarkers.

## 1. Introduction

Proteins display multiple functional and structural features in cellular machinery. They are also essential elements as indicators and predictors of diseases through their inherent structural properties, concentration, and 3-D orientation [1]. Such a compound, hemoglobin, a tetrameric protein in red blood cells, consists of two dimer subunits with an iron-carrying protein that transports oxygen and carbon dioxide throughout the body and also maintains acid-base balance in the blood. Any structural and concentration changes in hemoglobin may lead to serious diseases, mostly caused by genetic factors, including hemoglobinopathies, thalassaemia and sickle cell anemia [2,3]. Although there are many laboratory-based analytical tools to determine hemoglobin levels, considerable limitations including expensive assays, multi-step procedures, long assay times, the need for skilled personnel, poor test stability and specificity still limit their implementations in resource-constrained settings [4]. Even though many efforts are directed at developing sensitive detection strategies, especially affinity-based methods, transportation, refrigeration, and storage are main issues in these regions, thereby easy-to-use, reliable, sensitive, durable, specific and long-term stable detection assays are urgently needed [5].

Besides affinity-based assays, molecular imprinting methods have attracted researchers due to their nanolevel molecular replicability of template/target proteins [6,7,8]. The molecular imprinting method is one of the fascinating surface modification technique that utilizes templates to create specific cavities for recognition of targets in a polymeric matrix. This method provides a broad range of versatility to imprint targets with different molecular size, three dimensional structure, and physicochemical properties. In contrast to biological molecules such as antibodies that require labor intensive, high-cost production and multiple quality-control procedures, molecularly imprinted polymers offer notable advantages including long-term stability, high durability in harsh conditions (e.g., high pressure and temperature), ease-of-preparation, versatility, reusability, inexpensive production, and high sensitivity to the target protein. Therefore, these molecular replica nanofilms produced by molecular imprinting methods are vital candidates for bio-assays in resource-constrained settings.

In this study, we designed a specific, sensitive and stable molecularly imprinted nanofilm on-chip to detect hemoglobin. As a detector, surface plasmon resonance technology was selected since it enables one to record minute binding events at the close vicinity of the surface [9]. Hemoglobin was utilized as a model protein for the molecular fingerprinting process. First, hemoglobin and acrylamide pre-complex was prepared with template and monomer mixture, and then the cross-linker (methylenebisacrylamide) was added to the pre-complex mixture to form a final mixture for polymerization. After addition of an initiator and activator (ammonium persulfate and tetramethyl ethylenediamine) pair to the final mixture, the whole mixture was then used to decorate to the molecularly imprinted nanofilm on-chip. The molecularly imprinted nanofilm on-chip was characterized and then the detection performance was investigated through kinetic parameters and isotherm models. The selectivity, reusability and storage stability performance of the molecularly imprinted nanofilm on-chip was also studied. Therefore, we developed an alternative strategy to monitor protein biomarker levels for clinical laboratories or primary care clinics with limited resources that are deprived of expensive infrastructure, skilled personnel, and storage/refrigeration.

## 2. Materials and Methods

### 2.1. Materials

Acrylamide (A3553, Sigma-Aldrich, St. Louis, MO, USA), N,N-methylenebisacrylamide (M7279, Sigma-Aldrich, St. Louis, MO, USA), ammonium persulfate (A3678, Sigma-Aldrich, St. Louis, MO, USA), N,N,N′,N′-tetramethylethylene diamine (T9281, Sigma-Aldrich, St. Louis, MO, USA), bovine hemoglobin (Hb, H3760, Sigma-Aldrich, St. Louis, MO, USA), bovine serum albumin (BSA, A2153, Sigma-Aldrich, St. Louis, MO, USA), lysozyme (Lyz, 62971, Sigma-Aldrich, St. Louis, MO, USA), transferrin (Trf, T8158, Sigma-Aldrich, St. Louis, MO, USA), myoglobin (Myb, M0630, Sigma-Aldrich, St. Louis, MO, USA), 2-propene-1-thiol (allyl mercaptan, 06030, Sigma-Aldrich, St. Louis, MO, USA), dipotassium hydrogen phosphate (K_2_HPO_4_, 60356, Sigma-Aldrich, St. Louis, MO, USA), potassium dihydrogen phosphate (KH_2_PO_4_, 04243, Sigma-Aldrich, St. Louis, MO, USA) and gold surfaces (GWC-1000-050, GWC Technologies, Madison, WI, USA) were purchased for all experiments.

### 2.2. Modification and Preparation of the NanoFilms

The modification of the nanofilm was first carried out by using allyl mercaptan due to the improvement of the coupling for the imprinting process [10]. Allyl mercaptan was dropped on the gold chip surfaces and incubated overnight to obtain pendant vinyl groups. After the modification, the gold chip surfaces were washed with ethyl alcohol and dried at room temperature for the imprinting process. The pre-complex was prepared with the model protein hemoglobin and different amounts of acrylamide monomer dissolved in a water and were stirred 30 min. The optimized ratio of hemoglobin to acrylamide was evaluated by UV-visible spectrophotometer (Model 1601, Shimadzu, Kyoto, Japan). The measurements were repeated until no increment of absorbance belonging to the formed pre-complex between hemoglobin and acrylamide was observable.

The molecularly imprinted nanofilm on-chip was prepared using the pre-complex and N,N-methylenebisacrylamide (6.0 mg) as a cross-linker. Then, 10 µL of ammonium persulfate (10%) and 10 µL of N,N,N′,N′-tetramethylethylenediamine (5%) as an initiator and activator pair were added to the monomer mixture to uniformly coat the modified chip surface by spin coating. The polymerization was accomplished under UV light (100 W, 365 nm) for 30 min. The unreacted monomer and impurities were removed by employing ethyl alcohol, followed by drying. The molecularly imprinted nanofilm on-chip was treated with desorption agent (0.01 M NaCl) for an hour to remove the template from the hemoglobin specific cavities of the chip. The washing steps were repeated several times until no band was observed at 406 nm by UV-visible spectrophotometry. A non-imprinted nanofilm on-chip was also produced by the same procedure without using hemoglobin.

### 2.3. Characterization of the NanoFilms

Characterization studies of the non-imprinted and molecularly imprinted nanofilms were done via atomic force microscope and ellipsometry analysis. The atomic force microscope analysis was employed in tapping mode (Nanomagnetics Instruments, Oxford, UK). The atomic force microscope system could perform measurements in high resolution due to the cantilever interferometer. The non-imprinted and molecularly imprinted nanofilm on-chips were placed to the atomic force microscope system by using double-sided carbon strip and images were obtained with oscillation frequency (341.30 Hz), vibration amplitude (1 voltage root mean square) and free vibration amplitude (2 voltage root mean square). Ellipsometry analysis of the non-imprinted and molecularly imprinted nanofilms was carried out by using an auto-nulling imaging ellipsometry system (Nanofilm EP3, Goettingen, Germany). A four-zone auto-nulling procedure integrating sample areas (50 µm × 50 µm) was followed by a fitting algorithm in the layer thickness analysis. All ellipsometry analyses were performed at a wavelength of 532 nm with an angle of incidence at 72° and were also carried out three times. The thickness results were reported as the mean value of the analysis plus standard deviations.

### 2.4. Kinetic Analysis

Kinetic analysis was first performed using in the same hemoglobin concentration (0.1 mg mL^−1^) in solutions of different pH that was changed from 4.0 to 8.0 and then with different hemoglobin concentrations that changed from 0.0005 mg mL^−1^ to 1.0 mg mL^−1^ in the solution (pH 6.0) that gave the maximum response with the same flow rate (150 µL min^−1^). The resonance angle and plasmon curve were adjusted and then the adsorption solution (pH 6.0) was passed from the surface plasmon resonance system to obtain a baseline. Then, the hemoglobin solutions were interacted with chips, individually. The change in reflectivity (%Δ*R*) was observed immediately and desorption agent, 0.01 M NaCl in deionized water, was supplied when the surface plasmon resonance system was in an equilibrium condition. After the desorption process, the chips were regenerated with ultrapure water and then adsorption solution was used for re-equilibration. These adsorption-desorption-regeneration steps were repeated for each sample solution. The signal changes for each sample concentration were then calculated from a series of difference images obtained by subtracting the reference image from the image obtained at each concentration of the hemoglobin. The reflectivity changes were then plotted versus concentration of the hemoglobin.

### 2.5. Selectivity, Reusability and Storage Stability Analysis

The selectivity analysis of the non-imprinted and molecularly imprinted nanofilm on-chips was investigated by using lysozyme (Lyz), transferrin (Trf), bovine serum albumin (BSA), and myoglobin (Myb) sample solutions as competing agents in same concentrations (0.1 mg mL^−1^). In addition, the selectivity properties were also done with a mixture of these competing proteins and hemoglobin. The total concentration of protein mixture was adjusted to 0.1 mg mL^−1^ at pH 6.0. To demonstrate the reusability performance of the molecularly imprinted nanofilm on-chip, different concentrations of hemoglobin solution ranging from 0.1 to 1.0 mg mL^−1^ were used, respectively. The molecularly imprinted nanofilm on-chip was also examined with the same hemoglobin concentration (0.1 mg mL^−1^) at different times (0, 3 and 27 months) to determine storage stability performance.

## 3. Results and Discussions

### 3.1. Preparation and Characterization of the NanoFilms

**Figure 1:**
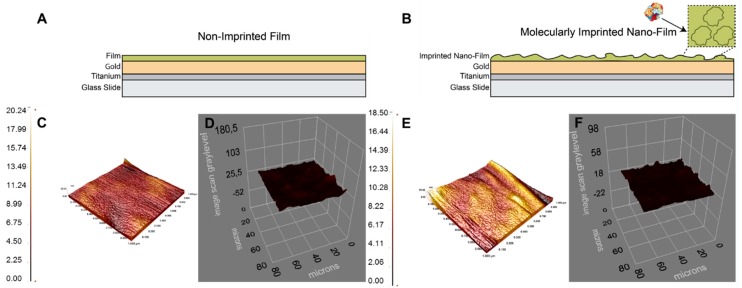
Schematic illustrations, atomic force microscope and ellipsometry images of the non-imprinted (**A**,**C**,**D**) and molecularly imprinted (**B**,**E**,**F**) nanofilms.

The pre-complex was prepared with the ratio 1 µmol:4 mmol of hemoglobin and acrylamide and the absorbance intensity increment ended up at this ratio. After obtaining an optimum ratio, the non-imprinted and molecularly imprinted nanofilms for hemoglobin molecule fingerprints were prepared as shown in Figure 1A,B. The atomic force microscope images of the bare, non-imprinted and molecularly imprinted nanofilm on-chips revealed that the average surface roughness values were increased from 0.54 nm to 1.05 nm from bare to non-imprinted nanofilm. After hemoglobin imprinting, the average surface roughness value was also increased to 1.86 nm, pointing out the success of the polymerization by the increase of roughness values. In addition, the root means square values were amplified by 3.4-fold (0.73 nm to 2.46 nm) after the imprinting process (Figure S1 and Figure 1C–E). To evaluate surface thickness after the polymerization and template removal, ellipsometry analysis was also performed. The surface thicknesses were calculated as 88.3 ± 3.3 nm and 87.9 ± 1.6 nm for non-imprinted and molecularly imprinted nanofilms, respectively (Figure 1D–F). These results were also coherent with the results obtained from the atomic force microscopy analysis. In sum, monolayer and almost homogeneous nanofilm formation after the polymerization process was successful.

### 3.2. Kinetic Analysis

To evaluate the kinetic behavior of the molecularly imprinted nanofilm on-chip, the binding of hemoglobin molecules was monitored using a surface plasmon resonance system that considerably reduces assay time, cost, and the need for labelling process. In addition, specific molecular interactions and binding events between molecularly imprinted nanofilm and hemoglobin can be measured in real-time, making the analysis quantitative while also determining the kinetic parameters.

In this analysis, the molecularly imprinted nanofilm on-chip was first washed with adsorption solution (pH 6.0) for 200 s to obtain a baseline, and then, hemoglobin solutions with different concentrations were introduced into the surface plasmon resonance system (SPR imager II, GWC Technologies, Madison, WI, USA) for 1000 s. Following this step, desorption buffer (0.01 M NaCl in deionized water) was applied for 200 s. After that, a second wash step was applied with the desorption buffer for 30 min, followed by washing with deionized water for 30 min and the adsorption buffer for 30 min. The same flow rate (150 µL min^−1^) was used for all experiments.

**Figure 2:**
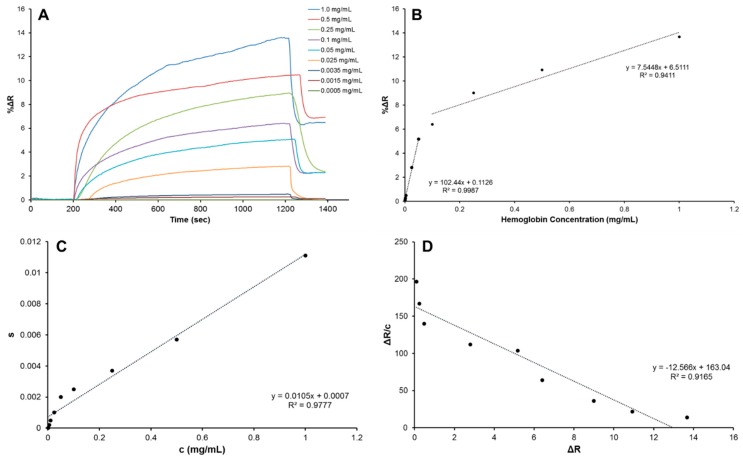
The combination of real-time responses (**A**), the calibration curve of molecularly imprinted nanofilm on-chip response to hemoglobin sample solutions (**B**), association kinetics analysis (**C**) and equilibrium analysis (**D**).

In addition, a rise in hemoglobin concentration produced an increase in the molecularly imprinted nanofilm response (Figure 2A). As referred earlier, hemoglobin sample solutions were prepared with pH 6.0 phosphate buffer. To obtain a maximum response, the effect of pH of the adsorption solution was investigated by using different pHs in a range from 4.0–8.0. As shown in Figure S2, the highest response was observed at pH 6.0. Acrylamide monomer can supply several H-bonding spots for hemoglobin template at this pH. Thus, the hemoglobin molecule can be polymerized in the polymeric matrix through the H-bond between the acrylamide amide group and the hemoglobin amino and carboxyl groups.

In the surface plasmon resonance system, the data was obtained as a change in reflectivity, %Δ*R*, demonstrating the absolute physical unit of measurement. In the measurements, %Δ*R* values in resonance frequency reached a plateau value around 1200 s. The relationships between hemoglobin concentration and %Δ*R* were obtained with this concentration range and the calibration curves were demonstrated in Figure 2B. In all solutions, the molecularly imprinted nanofilm on-chip extended the plateau value up to 1200 s (~23 min), and increments in hemoglobin concentrations resulted in higher %Δ*R* values. After applying the desorption buffer to the system, %Δ*R* values decreased to approximately the initial value. Therein, the %Δ*R* values increased from 0.098 to 13.67 between 0.0005 mg mL^−1^ and 1.0 mg mL^−1^ of hemoglobin concentrations. Further examinations on the performance of the molecularly imprinted nanofilm on-chip indicated a high (99%) precision for hemoglobin concentrations from 0.0005 to 0.05 mg mL^−1^ with an equation y = 102.44x + 0.1126, as well as 94% precision was observed between 0.1 and 1.0 mg mL^−1^ with an equation y = 7.5448x + 6.5111. Theoretical calculation results (using the 3S/b formula, where S is the standard deviation of the chip response can be estimated by the standard deviation of either y-intercept of regression lines) showed that the molecularly imprinted nanofilm on-chip was able to detect as low as 0.00035 mg mL^−1^.

All hemoglobin concentrations produced a response in the surface plasmon resonance measurements. In the pseudo first order reactions, the hemoglobin concentration was kept constant and the binding events were defined by Equation (1):

(1) $$\frac{d\Delta R}{dt}=k_{a}c\left(\Delta R_{max}-\Delta R\right)k_{d}\Delta R$$

where *d*Δ*R*/*dt* is the rate change of the molecularly imprinted nanofilm response; Δ*R* and Δ*R_max_* are the measured value and maximum response calculated, respectively; *c* is hemoglobin concentration; and *k_a_* and *k_d_* are association and dissociation rate constants, respectively. The association constant (*K_A_*) was calculated using *K_A_* = *k_a_*/*k_d_*. At the equilibrium (*d*Δ*R*/*dt* = 0), the equation was then revised as Equation (2):

(2) $$\Delta R_{eq}/c=K_{A}\Delta R_{max}-K_{A}\Delta R_{eq}$$

The *K_A_* value was also obtained using the plot of Δ*R_eq_*/*c* vs. Δ*R_eq_*, and the dissociation constant, *K_D_*, was calculated as 1/*K_A_*. Further derivations of Equation (1) were revised to Equation (3):

(3) $$\frac{d\Delta R}{dt}=k_{a}c\Delta R_{max}-\left(k_{a}c+k_{d}\right)\Delta R$$

The plot of *d*Δ*R*/*dt* versus Δ*R* provided a straight line with a slope of −(*k_a_* + *k_d_*). With the identification of Δ*R_max_*, both *k_a_* and *k_d_* were calculated from an association sensorgram [11]. A preferred method is to quantify the association sensorgram at various hemoglobin concentrations [12]. The forward and backward reaction rates were obtained from a plot of *d*Δ*R*/*dt* versus Δ*R*, and the value of s (the slope) was calculated using the Equation (4):

(4) $$s=k_{a}c+k_{d}$$

The plot of *s* versus *c* provided a straight line and *k_a_* was defined as the slope of this plot. In principle, the intercept on the ordinate presents *k_d_* [13]. The dissociation can also be quantified using Equation (5):

(5) $$ln\left(\Delta R_{0}/\Delta R_{t}\right)=k_{d}\left(t-t_{0}\right)$$

where Δ*R*_0_ is the initial response at *t*_0_; Δ*R* and *t* are calculated from the dissociation curve [14]. Association kinetics analysis graph was shown in Figure 2C. Equilibrium analysis, i.e., Scatchard, is employed to evaluate the experimental data for reversible host/guest interactions and identify the total binding sites the host has at the equilibrium condition [15].

(6) $$\Delta R_{ex}/c=K_{A}\left(\Delta R_{max}-\Delta R_{eq}\right)$$

The equilibrium analysis graph was also presented in Figure 2D. According to the kinetic calculations and analysis, high correlation coefficients of the experimental data were observed, with values of 0.92 and 0.98 for the equilibrium and association kinetics analysis. In the equilibrium analysis, the *K_A_* and *K_D_* coefficients were calculated as 12.6 mL mg^−1^ and 0.08 mg mL^−1^, respectively. In the association kinetic analysis, the *K_A_* and *K_D_* coefficients of 15 mL mg^−1^ and 0.07 mg mL^−1^, were found, respectively.

### 3.3. Adsorption Isotherm Models

The adsorption isotherm models identify multiple parameters, including detection capability, selectivity and surface homogeneity. As stated in Equations (7) and (8), the binding events between the molecularly imprinted nanofilm on-chip and hemoglobin molecules were defined with two different adsorption isotherm models as follows:

(7) $$\mathrm{Langmuir}\;\Delta R=\{\Delta R_{max}[c]/K_{D}+[c]\}$$

(8) $$\mathrm{Freundlich}\;\Delta R=\Delta R_{max}{\left[c\right]}^{1/n}$$

**Figure 3:**
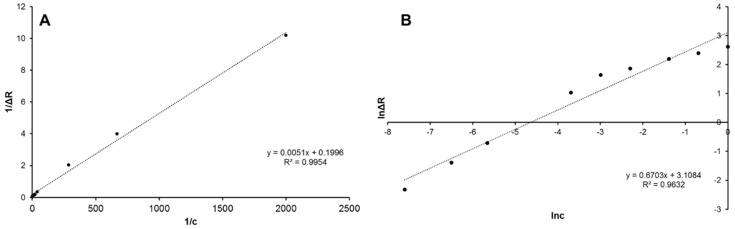
Adsorption isotherm models: Langmuir (**A**) and Freundlich (**B**).

Langmuir adsorption isotherm model is based on the acceptance of a homogeneous distribution of equal energy (without extra interactions), whereas the Freundlich adsorption isotherm model demonstrates heterogeneous interactions. The heterogeneity index, 1/*n*, ranges between 0 and 1. As heterogeneity decreases, 1/*n* becomes closer to 1, and equals to 1 for a homogeneous system [16]. Experimental data were plotted according to the above equations of adsorption isotherm models (Figure 3A,B). As a result, the experimental data were fitted well to the Langmuir adsorption isotherm model with high correlation coefficient (*R*^2^ = 0.99) compared to the Freundlich adsorption isotherm model, which means that the binding of hemoglobin molecules onto the molecularly imprinted nanofilm on-chip is a monolayer on a homogeneous surface, and *K_A_* and *K_D_* coefficients were found as 39.1 mL mg^−1^ and 0.03 mg mL^−1^, respectively. All coefficients were provided in Table 1.

### 3.4. Selectivity Analysis

In the selectivity analysis, the responses of potential competitors (i.e., lysozyme (Lyz), transferrin (Trf), bovine serum albumin (BSA), and myoglobin (Myb)) were evaluated for both non-imprinted and molecularly imprinted nanofilm on-chips. The concentrations of competitive protein solutions were prepared as 0.1 mg mL^−1^.

In addition, the selectivity coefficient (*k*) was also calculated according to Equation (9). The relative selectivity coefficient (*k*′) was depicted using the Equation (10):

(9) $$k=\Delta R_{template}/\Delta R_{competitor}$$

(10) k′=kmolecularly imprintedknon−imprinted

**Figure 4:**
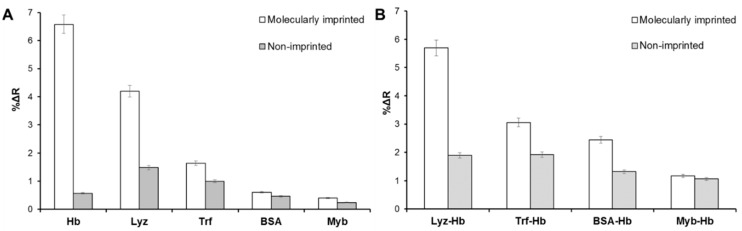
The real-time responses of the non-imprinted and molecularly imprinted nanofilm on-chips in a single protein solution (**A**) and protein mixture solutions (**B**).

All selectivity and relative selectivity coefficients of the non-imprinted and molecularly imprinted nanofilm on-chips were calculated and demonstrated in Table 2. The molecularly imprinted nanofilm on-chip provided higher %Δ*R* values than those of non-imprinted nanofilm on-chip (Figure 4A,B). Furthermore, Lyz exhibited higher binding than the other competitive proteins as the molecular weight of Lyz (MW: 14.3 kDa) is much lower than BSA (MW: 66 kDa) and Trf (MW: 76 kDa). In principle, small proteins can diffuse easily to cavities and generate non-specific binding. Contrary to the small proteins, large proteins can be easily removed from cavities due to steric hindrance [17]. Although the molecular weight of Myb (MW: 17 kDa) is close to Lyz, amino acid sequence and 3-D orientation of Myb is distinct, resulting in lower non-specific binding. In addition, a mixture of protein solutions (Lyz-Hb, Trf-Hb, BSA-Hb, and Myb-Hb) with same concentrations (0.1 mg mL^−1^) was evaluated to compare selectivity performance. The protein mixture of Lyz-Hb showed the highest response to the molecularly imprinted nanofilm on-chip. On the other hand, protein mixtures resulted in lower responses on the non-imprinted nanofilm on-chip. Overall, structural memory and specificity were only observed in molecularly imprinted nanofilm on-chip and also the molecularly imprinted nanofilm on-chip had particular detection capability toward hemoglobin because of the hemoglobin specific imprinting process.

The comparison of the real-time responses of non-imprinted and molecularly imprinted nanofilm on-chips is also shown in Figure S3. Three sample solutions with different hemoglobin concentrations (0.05, 0.1 and 0.25 mg mL^−1^) were prepared and applied to non-imprinted and molecularly imprinted nanofilm on-chips, separately. As clearly seen that the molecularly imprinted nanofilm on-chip has high responses to the hemoglobin molecules of different concentration due to the fact that there are no specific cavities for hemoglobin molecules to bind in the non-imprinted nanofilm on-chip.

### 3.5. Reusability and Storage Stability Analysis

**Figure 5:**
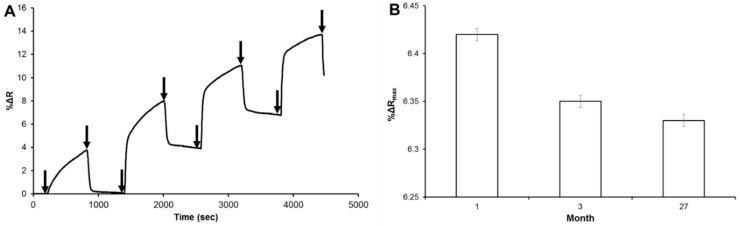
Reusability (**A**) and storage stability (**B**) of the molecularly imprinted nanofilm on-chip.

The four adsorption-desorption cycles that shown by arrows were employed to test the reusability of the molecularly imprinted nanofilm on-chip. As seen in Figure 5A, the hemoglobin solutions were prepared in different hemoglobin solutions (0.05, 0.25, 0.5 and 1.0 mg mL^−1^) and then applied consecutively to the molecularly imprinted nanofilm on-chip, and the real-time responses were increased according to the increasing hemoglobin concentration. In addition, the molecularly imprinted nanofilm on-chip was tested with the same hemoglobin concentration (0.1 mg mL^−1^) at different times (0, 3, 27 months) to show storage stability. One of the most significant challenges in this field is to keep the bio-sensing layer stable for long-term storage. However, most bio-sensing systems notably face this obstacle since antibody and protein-based layers have difficulties to prolong their stability at room temperature, and these layers hence require refrigeration at certain temperatures (ranging from +4 °C to −80 °C) according to their stability capability. Even under refrigeration conditions, the timeframe is around one month at 4 °C and can be extended with chemical treatments up to one year at −80 °C [18]. Although some studies have reported that antibodies could be stored around a year in liquid nitrogen, sensor surfaces cannot resist such harsh conditions, resulting in function problems on the chip surface. Therefore, ‘3 months’ was designated as the first (starting) stability control check to evaluate first performance efficiency after storage at room temperature. Recently, the researchers demonstrated that antibody-coated chips could be stored at room temperature after they were evacuated and treated with a certain concentration of trehalose solution [19]. This procedure kept the chip performance and antibody stability constant for 24 weeks. To evaluate stability performance of the molecularly imprinted nanofilm on-chip during longer periods (more than 6 months), 27 months was determined as a second stability check point. Such a long period of storage at room temperature did not result in any statistically significant change in our chip performance. The response of the molecularly imprinted nanofilm on-chip was decreased from 6.42% to 6.33% and the performance loss was only 0.09% in 27 months (Figure 5B). Finally, the comparison of the different systems for hemoglobin detection is summarized in Table 3. The table was prepared with different parameters such as detection system and range, limit of detection, selectivity, reusability year by year since 2013 and based on molecular imprinting polymer database references.

## 4. Conclusions

In this study, molecular fingerprints of hemoglobin were obtained on a nanofilm chip through the molecular imprinting strategy. The as-prepared chip successfully detected hemoglobin levels down to 0.0005 mg mL^−1^ without any significant changes in its specificity after periods as long as several months. Due to durability and stability shortages in affinity-based assays, resource-constrained settings are still seeking for alternative strategies allowing long-term storage without refrigeration while keeping the performance of assays constant. Owing to the physical and chemical robustness, low-cost production, reusability, high selectivity, durability, and storage stability of the presented nanofilm on-chip hold we can claim a pivotal improvement over currently available detection strategies in these settings. In addition, the versatility and capability of molecularly imprinted methods will potentially enable one to tailor these nanofilms for detecting other protein markers and cells, as well as integrate them with different detector systems in the future.

## Figures and Tables

**Table 1:** All coefficients of kinetic analysis.

Equilibrium Analysis		Association Kinetic Analysis		Langmuir		Freundlich	
Δ*R_max_*	13	*k_a_*, mL mg s^−1^	0.011	Δ*R_max_*	5.01	Δ*R_max_*	22.4
*K_A_*, mL mg^−1^	12.6	*k_d_*, s^−1^	0.001	*K_D_*, mg mL^−1^	0.03	1/*n*	0.67
*K_D_*, mg mL^−1^	0.08	*K_A_*, mL mg^−1^	15	*K_A_*, mL mg^−1^	39.1	*R* ^2^	0.96
*R* ^2^	0.92	*K_D_*, mg mL^−1^	0.07	*R* ^2^	0.99		
		*R* ^2^	0.98				

**Table 2:** The selectivity and relative selectivity coefficients of non-imprinted and molecularly imprinted nanofilm on-chips.

Protein	Molecularly Imprinted		Non-Imprinted		
	%∆*R*	*k*	%∆*R*	*k*	*k*′
Hb	6.58		0.57		
Lyz	4.19	1.57	1.48	0.39	4.08
Rrf	1.64	4.01	1	0.57	7.04
BSA	0.61	10.79	0.46	1.24	8.71
Myb	0.4	16.45	0.24	2.38	6.93

**Table 3:** The comparison of different detection systems with this study for hemoglobin detection.

Detection System	Base on	Detection Range	Limit of Detection	Selectivity	Reusability	Time	Ref.
Electrochemical	Magnetic nanoparticles	0.005–0.1 mg mL^−1^	0.001 mg mL^−1^	Lyz, BSA, HRP	Not reported	7 min	[20]
Fluorescence	Core-shell	0.02–2.0 µM	6.3 nM	Lyz, BSA, OB	5 times	15 min	[21]
Localized surface plasmon resonance	Artificial antibody	0.5–20 µg mL^−1^	Not reported	HSA, BSA, Lyz	Not reported	120 min	[22]
Differential pulse voltametry	eATRP	1.10^−10^–1.10^1^ mg L^−1^	7.8.10^−11^ mg L^−1^	Lyz, BSA, HSA, IgG	3 times	120 min	[23]
Fluorescence	Gold nanoparticle	0.1–20 µmol L^−1^	0.03 µmol L^−1^	BSA, BHb, Alb, CE	Not reported	Not reported	[24]
Differential pulse voltametry	Cryogel	1.10^−8^–1.10^2^ mg L^−1^	6.7.10^−9^ mg L^−1^	Lyz, BSA, HSA	3 times	120 min	[25]
Electrochemical	SAM	1–20 µg mL^−1^	Not reported	Myb	Not reported	Not reported	[26]
Localized surface plasmon resonance	PEGlated nanorattle	1–2500 ng mL^−1^	Not reported	HSA, BSA, Myb	Not reported	Not reported	[27]
Chemiluminescent	Carbon nanotube	5.10^−10^–7.10^−7^ mg mL^−1^	1.5.10^−10^ mg mL^−1^	BSA, Lyz	Not reported	Not reported	[28]
Electrochemical	Gold nanoparticle	1.10^−11^–1.10^−2^ mg mL^−1^	Not reported	BSA, EA, Lyz	5 times	Not reported	[29]
Electrochemical	Nanoparticle	0.005–0.1 mg mL^−1^	25.8 ng mL^−1^	Lyz, HRP	Not reported	10 min	[30]
Electrochemical	Graphene-carbon electrode	1.10^−10^–1.10^−3^ mg mL^−1^	3.09.10^−11^ mg mL^−1^	BSA, HSA, Lyz, ATP, BI	3 times	120 min	[31]
Electrochemical	Magnetic nanoparticle	5.10^−7^–1.10^−5^ mg mL^−1^	1.184.10^−8^ mg mL^−1^	BSA, Lyz, Cyt C, HRP	Not reported	70 min	[32]
Electrochemical	Graphene composite	1.10^−9^–1.10^−1^ mg mL^−1^	2.10^−10^ mg mL^−1^	BSA, Lyz, EA, Pap	Not reported	10 min	[33]
Surface plasmon resonance-Electrochemical	Thin film	0.0005–5.0 mg mL^−1^	0.000435 mg mL^−1^	BSA, Lyz, Ova	Not reported	25 min	[34]
Electrochemi-luminescence	Magnetic nanocomposite	0.1–4.10^4^ pg mL^−1^	0.023 pg mL^−1^	BSA, CEA, AFP, HCG, HIgG	Not reported	70 min	[35]
Phosphorescence	Quantum dot	1.10^−7^–5.10^−6^ mol L^−1^	3.8.10^−8^ mol L^−1^	Not reported	11 times	15 min	[36]
Electrochemical	Gold microdentrites	0.1–4.10^3^ µg mL^−1^	0.05 µg mL^−1^	BSA, Lyz, Cyt C, Ova	5 times	60 min	[37]
Electrochemical	Quantum dot-carbon nanotube	27.8–444 ng mL^−1^	6.73 ng mL^−1^	BSA, Trp, Crp, Glu, Dop, Cys, AA, Ins	Not reported	Not reported	[38]
Fluorescence	Quantum dot	0.02–2.1 µM	9.4 nM	BSA, Lyz, OB	Not reported	60 min	[39]
Surface plasmon resonance	Nanofilm	0.0005–1.0 mg mL^−1^	0.00035 mg mL^−1^	Lyz, BSA, Trf, Myb	4 times	23 min	This study

## Supplementary Materials

The following are available online at http://www.mdpi.com/1424-8220/18/9/3016/s1.
