# Supplementary material for: Molecular Fingerprints of Hemoglobin on a Nanofilm Chip
Source: Sensors (Basel). 2018 Sep 9;18(9):3016. doi: 10.3390/s18093016 (PMC6165033; doi:10.3390/s18093016)
Supplement: Supplementary file 1 [file sensors-18-03016-s001.pdf]

*Supplementary Material*

# Molecular Fingerprints of Hemoglobin on a NanoFilm Chip

**Yeşeren Saylan and Adil Denizli ***

Department of Chemistry, Hacettepe University, 06800, Ankara, Turkey
* Correspondence: Hacettepe University, Department of Chemistry, 06800, Ankara, Turkey;
denizli@hacettepe.edu.tr

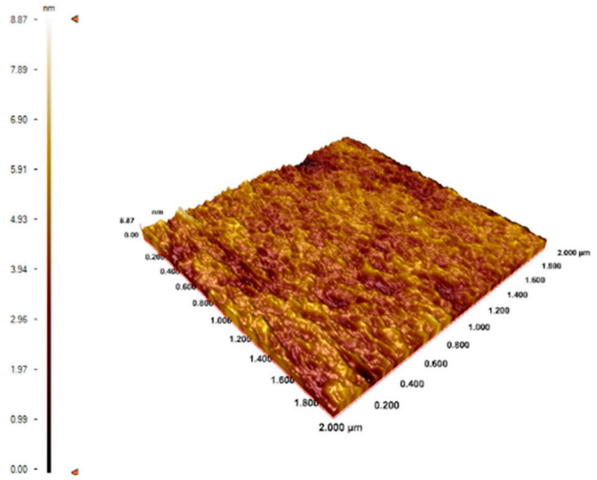


**Figure S1.** The atomic force microscope images of the bare chip.

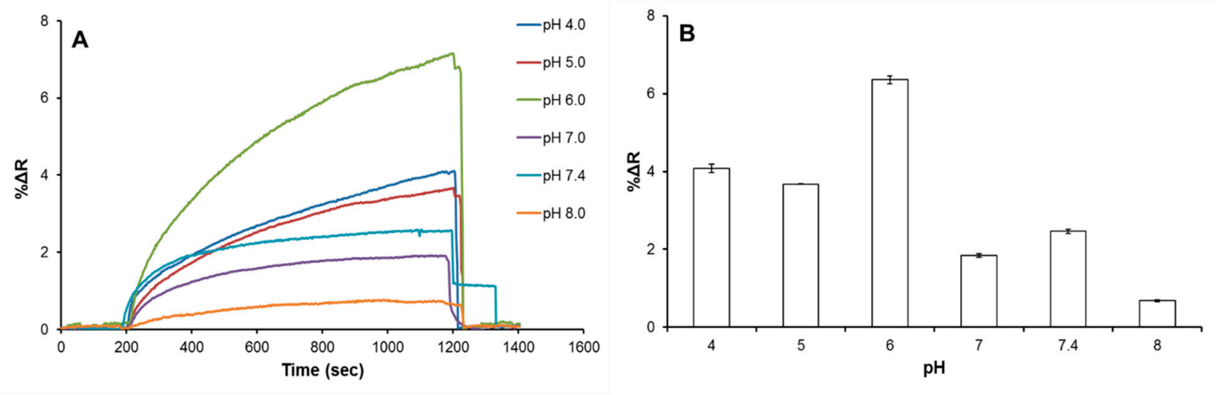


**Figure S2.** The real-time responses of molecularly imprinted nanofilm on-chip to same concentration of hemoglobin solutions at different pH in terms of a sensorgram (A) and a bar graph (B).

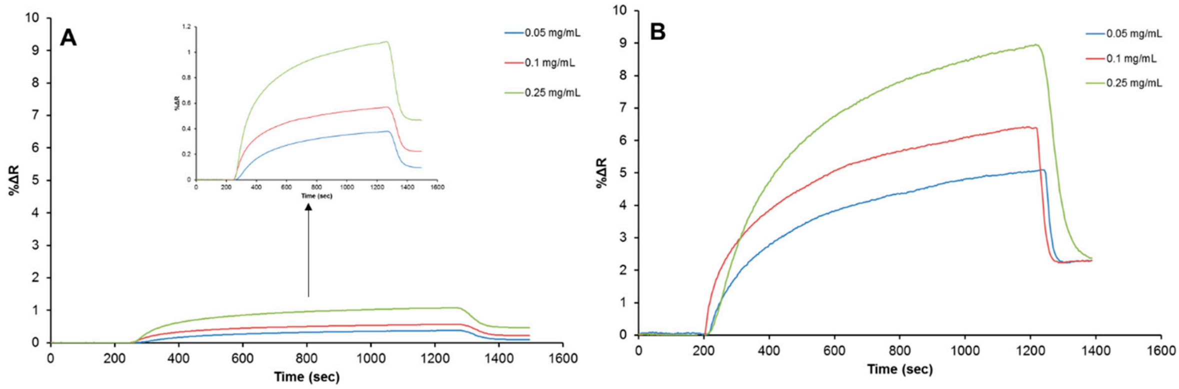


**Figure S3.** The comparison of real-time responses of non-imprinted (A) and molecularly imprinted nanofilm on-chips to hemoglobin sample solutions.
